# The pockets guide to HLA class I molecules

**DOI:** 10.1042/BST20210410

**Published:** 2021-09-28

**Authors:** Andrea T. Nguyen, Christopher Szeto, Stephanie Gras

**Affiliations:** 1 Department of Biochemistry and Genetics, La Trobe Institute for Molecular Science, La Trobe University, Bundoora, Victoria 3083, Australia; 2 Department of Biochemistry and Molecular Biology, Biomedicine Discovery Institute, Monash University, Clayton, Victoria 3800, Australia

**Keywords:** epitope presentation, HLA, HLA classification, peptide, peptide binding motif

## Abstract

Human leukocyte antigens (HLA) are cell-surface proteins that present peptides to T cells. These peptides are bound within the peptide binding cleft of HLA, and together as a complex, are recognised by T cells using their specialised T cell receptors. Within the cleft, the peptide residue side chains bind into distinct pockets. These pockets ultimately determine the specificity of peptide binding. As HLAs are the most polymorphic molecules in humans, amino acid variants in each binding pocket influences the peptide repertoire that can be presented on the cell surface. Here, we review each of the 6 HLA binding pockets of HLA class I (HLA-I) molecules. The binding specificity of pockets B and F are strong determinants of peptide binding and have been used to classify HLA into supertypes, a useful tool to predict peptide binding to a given HLA. Over the years, peptide binding prediction has also become more reliable by using binding affinity and mass spectrometry data. Crystal structures of peptide-bound HLA molecules provide a means to interrogate the interactions between binding pockets and peptide residue side chains. We find that most of the bound peptides from these structures conform to binding motifs determined from prediction software and examine outliers to learn how these HLAs are stabilised from a structural perspective.

## Introduction

The major histocompatibility complex (MHC) class I, known as human leukocyte antigen (HLA) class I in humans, is an essential surface molecule composed of a heavy chain and an invariant beta-2-microglobulin (β2m) that presents peptides to T cells [1]. HLA molecules are critical for the selection and activation of T cells and play a key role in the immune response to many pathogens.

There are two main classes of HLAs: HLA class I (HLA-I) and class II (HLA-II) with major structural differences between the two. Most prominently is that the HLA-I peptide binding cleft is closed at the N and C termini, and therefore, restricts the bound peptide to an optimal length of 8–10 amino acids (reviewed in [2]). Both ends of the binding cleft of HLA-II is open-ended, and thus, has a preference for peptides that are >13 amino acids in length [3].

The peptides presented by HLA molecules can be derived from host proteins (self-peptides) or from a pathogenic source such as viruses and bacteria. HLA is the most polymorphic molecule in humans, with >22 000 HLA alleles reported to date [4]. This allows HLAs to present a wide range of pathogenic-derived peptides to T cells (reviewed in [5]) and can also help to limit any pathogenic mutant escape from the immune system at a population level.

Due to this extreme diversity, each individual will present a different set of peptides, termed peptide repertoire, to their T cells, and this has greatly limited the use of T cell-based vaccines or therapeutics, as they are not applicable to a wide population. Despite this limitation, research continues to provide a better understanding of the relationship between pathogens, HLA presentation and peptide binding, which can dictate the T cell response. As CD8+ T cells are responsible for recognising peptide-bound HLA-I, killing infected cells and thus clearing infection, we have focused our review on HLA-I molecules.

The motivation behind characterising and understanding HLA binding pockets and their polymorphisms is to determine which peptides can bind to and be presented to the immune system. Interestingly, the polymorphisms that define each HLA variant have been found to impact peptide binding the most [6].

Although, there are many HLA alleles in existence, some alleles are more common in the population than others (e.g. HLA-A*02:01, HLA-A*24:02, HLA-B*35:01, etc.) and are often the subject of study for T cell based therapeutics. These HLA molecules have specificity for certain amino acid side chains in distinct binding pockets within the HLA binding cleft, which limits peptide sequences it can present to the immune system from any one pathogen.

Within the past two decades, over >600 structures of peptide-HLA (pHLA) complexes have been solved by X-ray crystallography and submitted to the Protein Data Bank (PDB). This allows insight into the binding patterns of peptides to specific HLA allotypes [7]. Interestingly, this still represents a very narrow slice of the diversity of HLA molecules (reviewed in [8]) but has been informative in understanding the rules of peptide binding specificity within specific HLA molecules.

## HLA pockets description

**Figure 1.**
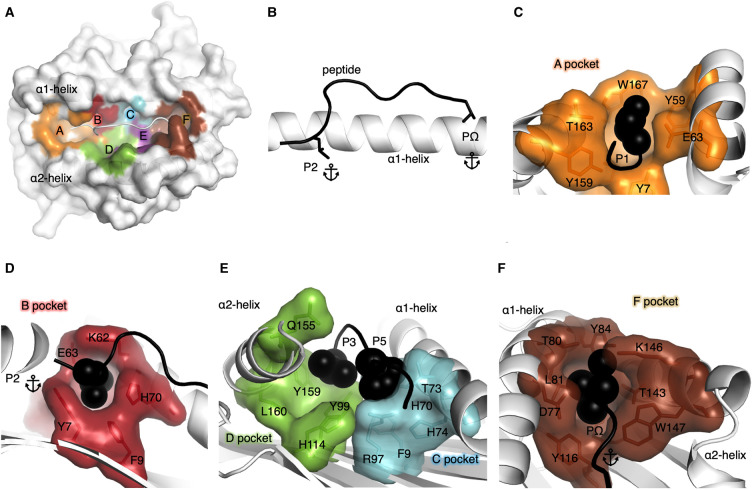
HLA pockets representation. (**A**) Surface representation of a HLA molecule (white) with the pockets within the cleft shown in different colours. Pocket A (orange), B (red), C (cyan), D (green), E (purple), F (brown) and the peptide shown as a cartoon in white. (**B**) Peptide represented as a black cartoon with primary anchor residues P2 and PΩ shown as stick against the α1-helix of the HLA shown as a white cartoon. (**C**) The A pocket of the HLA represented by an orange surface with the residues making up the pocket shown as orange sticks and the surrounding residues represented by a white cartoon. The P1 residue of the peptide is represented by black spheres nested into the A pocket. (**D**) The B pocket of the HLA represented by a red surface with the residues making up the pocket shown as red sticks and the surrounding residues represented by a white cartoon. The primary anchor P2 residue of the peptide is represented by black spheres. (**E**) The C pocket of the HLA represented by a cyan surface and residues as sticks, while the D pocket is represented by a green surface with the residues making up the D pocket shown as green sticks. The P3 and P5 of the peptide is represented by black spheres. (**F**) The F pocket of the HLA represented by a brown surface with the residues making up the pocket shown as brown sticks and the surrounding residues represented by a white cartoon. The PΩ of the peptide is represented by black spheres.

**Figure 2.**
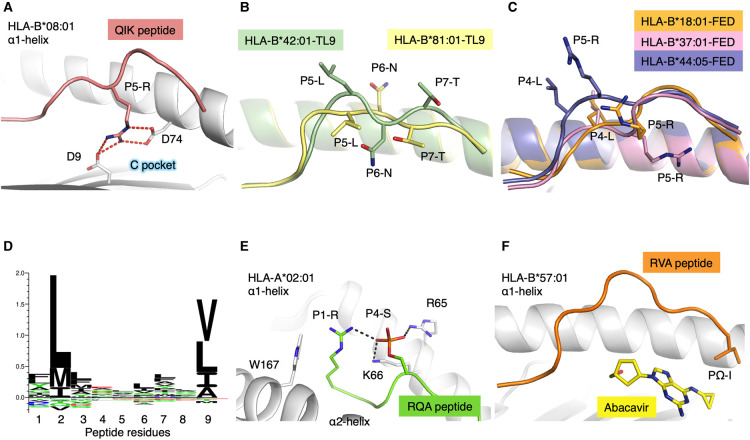
HLA polymorphism impacts on peptide presentation and binding. (**A**) The α1-helix of HLA-B*08:01 shown as a white cartoon with the residues interacting with the QIK peptide (salmon cartoon) shown as stick. The P5 of the QIK peptide is shown as stick, forming salt bridges (red dashed lines) with the Asp9 and Asp74 of the HLA molecule. (**B**) Overlay of HLA-B*42:01 (green cartoon) and HLA-B*81:01(yellow cartoon) each presenting the TL9 peptide, in a distinct conformation, shown as cartoon (green and yellow, respectively) with P5, P6 and P7 of the peptide shown as stick. (**C**) Overlay of HLA-B*18:01(orange cartoon), HLA-B*37:01 (pink cartoon) and HLA-B*44:05 (purple cartoon) presenting the FED peptide shown as cartoon (orange, pink and purple, respectively) with P4 and P5 of each peptide shown as stick. (**D**) Distribution of preferred peptide residues located into the B and F pockets of HLA-A*02:01 using Seq2logo2.0 [69]. (**E**) HLA-A*02:01 (white cartoon) with select residues shown as stick presenting the RQA peptide (green cartoon) with P1 shown as stick and P4 represented as an orange stick. The P4-S phosphorylated is represented as stick forming hydrogen bonds (black dashed lines) with the R65 from the HLA and the P1-R from the peptide. (**F**) HLA-B*57:01 (white cartoon) presenting the RVA peptide (orange cartoon) with the PΩ shown as stick, in the presence of Abacavir in the F pocket of the HLA (yellow stick).

HLA-I molecules possess six distinct binding pockets within the binding cleft termed as pockets A–F [9] (Figure 1), which allow peptide residue side chains to anchor or bind deeply within each pocket. Each HLA pocket has allotype-specific biochemical properties [10] based on the polymorphisms of each HLA allotype. The amino acid make-up of each binding pocket thus determines the peptide side chain specificity that can bind. Importantly, pockets B and F house the primary anchor residues of the peptide. These anchor residues form the main interactions between peptide and HLA, and is suggested to play a key role in stabilising the peptide-HLA complex [1,10–12]. In HLA-I, pocket B houses position 2 (P2) of the peptide, whilst pocket F usually accommodates the C-terminal residue of the peptide (PΩ) (Figure 1B). Here, we describe each of the six pockets that form the HLA binding cleft (Table 1), based on the structures of the HLA molecules obtained by X-ray crystallography. The A pocket is composed of 9 residues that traditionally helps bind the N-terminal group of the peptide, usually the first residue or P1, as well as forming the closed end or ‘wall' of the N-terminal part of the cleft (Figure 1C). Large aromatic residues are often present at position 59, 167, 171 (for e.g. in HLA-A*02:01) that helps close off this end of the cleft, which is a notable structural difference relative to HLA-II that has an open-ended conformation in their cleft. The closed conformation constrains the peptide length that is often shorter in HLA-I than HLA-II molecules, with some exceptions [2]. The B pocket consists of 10 residues (Table 1) with residues 9, 45, 63, 66, 67, 70, and 99 being the key residues that determine side chain specificity. The B pocket is binding one of the two primary anchor residues of the peptide, at P2, and determines the nature of the P2 residue able to bind to each HLA allotype (Figure 1D). The C and D pockets face each other in the middle of the cleft and are composed of 5 and 6 residues, respectively (Table 1). The C pocket is located against the α1-helix and the D pocket against the α2-helix of the HLA, both pockets bind secondary anchor residues at P3 and/or P5/6 depending on the peptide (Figure 1E). Some HLAs favour peptides with secondary anchor residues, for e.g. HLA-B*08:01 with a P5-R forming a salt bridge with Asp7 and Asp9 from the cleft (Figure 2A) [13]. The E pocket is formed by 5 residues and also partially binds the P5/6 secondary anchor residue as well as C-terminal residues of the peptide. The F pocket is composed of residues 77, 80, 81, 84, 95, 116, 123, 143, 146, and 147 (Figure 1F). Residues 77, 80, 81 and 116 are considered the key residues that determine the specificity of PΩ [10,12]. In addition, and as for pocket A, the last pocket also closes the C-terminal end of the cleft, with large aromatic residues at position 80, 81, 84, 123, 143 (as observed in HLA-A*02:01). The Tyr84 is conserved in about a third of HLA-I molecules [4], and replaced by Phe84 in ∼10% of HLA-I, and can be used as a switch that opens to enable the binding of longer peptides [14,15]. Interestingly, a large bulky residue at position 84 is also observed in lipid and metabolite antigen binding MHC-like molecules CD1 and MR1, respectively. The Tyr84 is shared in human CD1a, while CD1b has a Phe84, CD1c has a His84 [16], and MR1 has also a His84 at the end of the C-terminal part of the antigen binding cleft.

**Table 1:** Residues forming the HLA class I molecule pockets

Pocket	Residues	Role of the pocket
**A**	5, 7, 59, 63, 66, 159, 163, 167, 171	Wall of the N-terminal part of the binding cleft, bind P1 residue
**B**	7, 9, 24, 34, 45, 63, 66, 67, 70, 99	Bind primary anchor residue P2
**C**	9, 70, 73, 74, 97	Bind secondary anchor residue at P3 and P5/P6 when presents, face pocket D
**D**	99, 114, 155, 156, 159, 160	Bind secondary anchor residue at P3 and P5/P6 when presents, face pocket C
**E**	97, 114, 147, 152, 156	Overlap with C/D pockets and contact secondary anchor residue at P5/P6 when presents and the C-terminal part of the peptide
**F**	77, 80, 81, 84, 95, 123, 143, 146, 147	Bind primary anchor residue PΩ, wall of the C-terminal part of the binding cleft

Although it has been determined that the main anchor residues are necessary for binding, secondary anchors can also contribute significantly to overall binding as well (reviewed in [17,18]) and improve the overall stability of the peptide-HLA complex (pHLA).

## HLA supertypes classification

The structural features of HLA pockets brought about the concept of HLA supertypes, introduced in the 90's [19,20], which classified a number of HLA-A and HLA-B allotypes into 9 HLA-I supertypes [10] (Table 2). They analysed the key residues that made up the B and F pockets to understand the type of anchor residue that the pockets preferred and used this to group HLAs into clusters of supertypes. This provided an index for some of the first peptide and epitope-based approaches for vaccine development [21–23].

**Table 2:** HLA class I supertype classification

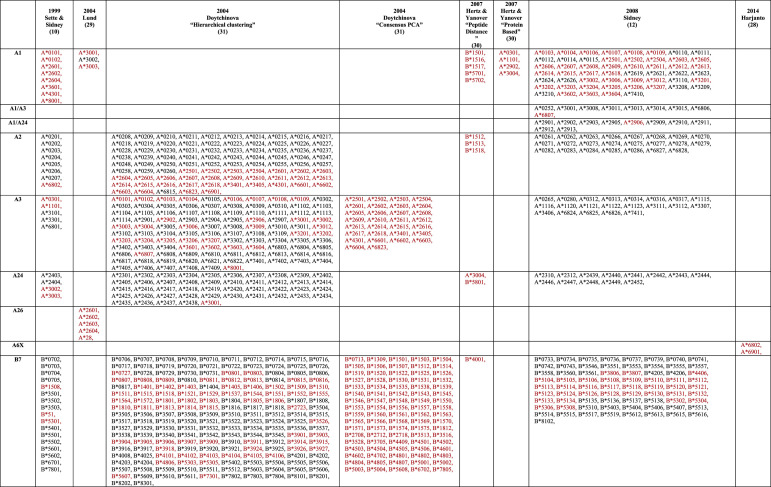
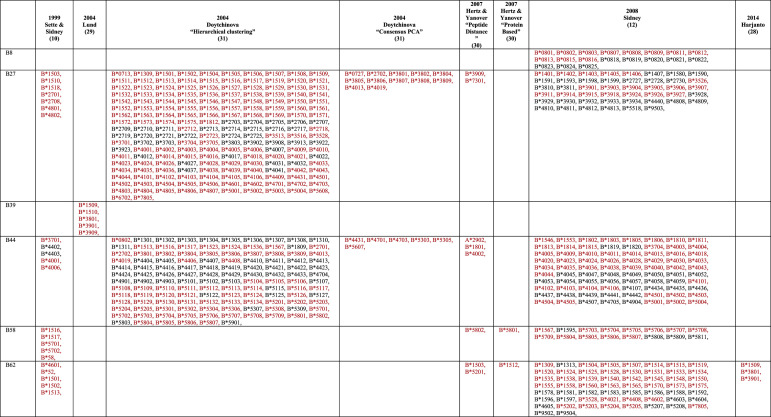

Alleles in red indicate an HLA allele that has changed supertype classification.

‘Consensus PCA' shows only alleles that are unique for ‘Consensus PCA' relative to ‘Hierarchical clustering' from [31].

‘Protein-based' shows only alleles that are unique for ‘Protein Based' allele classification relative to ‘Peptide Distance' from [30].

Theoretically, peptides that bind to one specific HLA within a supertype, would also likely bind to other HLAs in the same supertype due to sequence similarity of the HLA cleft. However, in practice, the classification is not always clear-cut, with many HLAs in the same supertype binding promiscuously to peptides of different supertypes [24–27]. Additionally, the use of this method requires each HLA to be classified within a HLA supertype. The work laid out by Sette & Sidney was further expanded to classify 750 HLA's in total, but by 2007 the number of HLA-I's that had been discovered had reached ∼1500 and now >22 000 [4]. This later rendition incorporated binding data into the HLA supertype classification and reshuffled some HLAs into different supertypes. Many different approaches used by several groups have yielded some new supertypes (for e.g. A6X supertype) or reshuffling of allotypes within existing supertype classifications [28–33] (Table 2).

Today, there are many clinical and immunological studies that use HLA supertypes as correlates or predictors of disease protection/severity [34–37], with current classifications incorporating up to 15 supertypes (Table 2). This highlights the simplicity and ease of use of supertypes to draw a relationship between HLA peptide restriction and disease outcome. In the context of peptide-based vaccines, peptide presentation targeted to a specific HLA supertype can give an indication of the potential coverage within a given population; however, we should be mindful that supertype classifications does not guarantee peptide binding in all their HLA denominations. In addition, if one peptide is binding to multiple HLA molecules from the same supertype it does not guarantee that those HLA will present the peptide in the same conformation, or that T cells will be able to recognise the peptide in the context of multiple HLA. For example, closely related HLA-B*42:01 and HLA-B*81:01 (belonging to the HLA-B7 supertype) both present the HIV derived Gag_180–188_ epitope (TPQDLNTML, TL9) but in very distinct conformations due to polymorphisms in the cleft [38] (Figure 2B). This is associated with different outcome between the HLA-B*42:01+ and HLA-B*81:01+ HIV+ patients [39]. Another example is HLA-B*37:01, HLA-B*18:01 and HLA-B*44:05 (belonging to the HLA-B44 supertype) each able to present the influenza epitope NP_338–346_ but in distinct conformations due to polymorphisms within the binding cleft [40] (Figure 2C). Here again, T cell activation was very different as the NP_338–346_ peptide is immunogenic only in the context of HLA-B*37:01 molecule. Therefore, there is no direct link between HLA molecules from the same supertype, binding the same peptide, and their ability to activate an immune response.

## MHC & peptide match prediction tools

To overcome a growing need for HLA peptide restriction, prediction software were made available online, which could predict binding between peptides and HLA molecules [41–46]. These early prediction tools devised algorithms that used peptide and HLA sequences to predict binding affinity based on peptide affinity data. Today, prediction tools (for e.g. NetMHCPan 4.1) combine both binding affinity data with mass spectrometry eluted peptides using machine learning strategies to create more powerful and accurate tools for prediction [5,47,48]. Earlier versions of MHC prediction tools struggled with availability of data [41,49], yet current versions benefit from large amounts of mass spectrometry data extracted from multiple HLA molecules simultaneously, where specificity for single HLAs is later determined [50,51].

MHC prediction embodies a bottom-up approach (peptide-based data), where numerous datasets of peptide binders from each HLA are used to predict binding. This contrasts with earlier concepts of supertypes, that embody a top-down approach (HLA sequence-based), where HLA amino acid sequence and polymorphisms determine peptide binders and thus supertype classification. Today, MHC prediction tools have found success in physicochemical approaches in T cell-based therapies such as peptide vaccine design that target specific HLAs [52,53], even though this was the proposed intention of HLA supertypes classification [10].

Another approach is to use epitope databases such as Immune Epitope Database (IEDB, www.iedb.org) to compile a list of peptides known to bind a given HLA, which also has the advantage of having all the information regarding immunogenicity of the peptides (T cell activation). This list of peptides can then be curated and a peptide binding motif can be generated from known and experimentally verified epitopes, and not just peptide binders. Of course, the limitation is that only a few HLA molecules are well represented in the database, and that the number of epitopes (peptides able to activate T cells) is less than the number of peptides able to bind a given HLA molecule [54].

## Comparison between structure-based and sequence based (binding data) methods

One of the initial hurdles for peptide prediction tools was the limitation of binding data [41,49]. However, as accessibility to both these became more widespread and their data more accessible, these results were able to be combined into improving HLA prediction software. Likewise, the number of pHLA structures has increased within the past two decades [8]. These pHLA structures provide empirical and definitive data of peptide restriction by HLAs displaying, in plain view, interactions within each pocket that define specificity [55].

As structural data continues to increase, so too can these data be used to train prediction tools to become more accurate [56,57]. Although knowledge of the specific interactions that influence peptide binding is expected to improve binding predictions [58,59], the development of structure-based methods have been slow relative to sequence based or binding data methods (reviewed in [56]).

Here, we investigated 14 HLA allotypes most frequently studied in structural biology or are a reference HLA within their supertype and compare their anchored residues with pocket specificities detailed in motif viewer [60] (Table 3). For HLA-A*02:01, 204 crystal structures have been solved in complex with various peptides free of T cell receptors (192 with complete peptide sequences). According to motif viewer [60], HLA-A*02:01 prefers L, M, or I in pocket B and V, L, I, or A in pocket F (Figure 2D). The majority of peptides conformed to these HLA-A*02:01 pocket B and F anchor preferences. For HLA-A*02:01, we found 46 out of 204 structures (23%) had bound peptides with an unpreferred anchor residue in pocket B or F. The majority of these (42/46) tolerated an unfavoured anchor at pocket B. Additionally, only one structure with both B and F pockets with unfavoured anchor residues was found for HLA-A*02:01. This structure showed HLA-A*02:01 in complex with a 10mer phosphopeptide (RQApSIELPSM) (PDB ID: 3BH8[61]), where P4-S is phosphorylated (Figure 2E). The crystal structures of several other phosphorylated P4-S sequences similar to this structure show a preference for a P1-R/P4-S motif as both P1-R and P4-S-phosphorylated side chains interact with each other and also form a network of interactions with W167 and R65 [61]. P2-Q fit poorly into the highly hydrophobic B pocket and was shown to form water-mediated contacts with Y99, whilst pushing the H70 side chain away from pocket B (both are key residues for pocket B specificity). Whilst PΩ-M caused the peptide main chain to be elevated to fit methionine's longer side chain into the smaller frame of HLA-A*02:01's F pocket. The authors hypothesised that even though P2-Q and PΩ-M are sub-optimally fit into pockets B and F, respectively, and would normally incur energetic penalties relative to more optimal anchor residues, these penalties in binding and peptide presentation are offset by phosphate-mediated interactions stabilising HLA-A*02:01. Interestingly, the peptide affinity between HLA-A*02:01 and the phosphorylated peptide is 159-fold stronger than that of the non-phosphorylated peptide sequence (i.e. RQASIELPSM) [61]. Mutations to P1 peptide or HLA R65 were shown to substantially decrease peptide binding affinity, demonstrating that these interactions were indeed contributing to the complex's high affinity [61].

**Table 3:** Peptide-HLA structures with unfavoured primary anchor residues

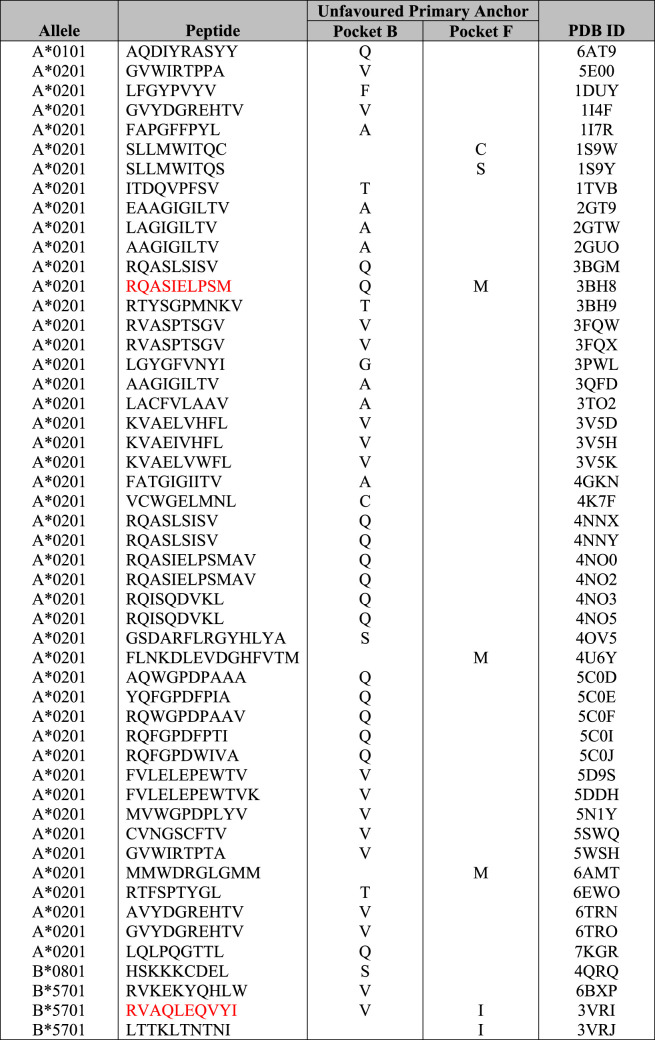

Peptides shown in red have both pockets B and F with unfavoured primary anchor residues.

Another outlier with unfavoured primary anchors was a self-peptide derived from ribonucleoprotein Sm D3, RVAQLEQVYI, in complex with HLA-B*57:01 (Table 3). Interestingly, this crystal structure was solved with abacavir, an antiretroviral used to treat HIV, binding into pockets C, D, E and F of HLA-B*57:01 (PDB ID: 3VRI [62]). Although abacavir made several contacts with HLA-B*57:01 pockets, there were limited interactions with abacavir and the peptide (only at PΩ-I), with P6-E and P7-Q peptide residues adopting a raised conformation outside of the binding cleft [62] (Figure 2F). Abacavir hypersensitivity syndrome is a HLA-associated drug reaction that exclusively affects individuals expressing the HLA-B*57:01 allele [63,64]. The abacavir-bound HLA-B*57:01 thus presents a new repertoire of peptides to the immune system, causing self-reactivity only in HLA-B*57:01+ individuals but not in HLA-B*57:02/03/11+ or HLA-B*58:01+ [62,65]. This highlights the allotype specificity of this drug hypersensitivity, where even single amino acid polymorphisms can result in altered HLA-drug interactions. Although this has been seen prominently in abacavir hypersensitivity syndrome, other drugs can cause similar reactions [66]. For example, another HIV antiretroviral drug, Nevirapine causes hypersensitivity reactions associated in a HLA-C cluster sharing a similar F pocket to HLA-C*04:01, which could indicate potential drug binding [67].

Therefore, there is a lot of plasticity in the way HLA pockets can bind residues or other small molecules, altogether providing insight into the broad repertoire of ligands that T cells recognise.

## Conclusion

HLAs are a key player in the immune system, being the primary target of T cells. Gaining a better understanding of their peptide binding capabilities will help inform researchers on the quality of the immune response to any given pathogen. The initial description of the HLA supertype families helped group a large and growing number of HLA molecules. However, as more data became available and the number of newly discovered alleles grew substantially, this classification needed to evolve and be revisited to remain relevant and useful. Today, over 22 000 HLA-I alleles have been discovered and classifying all these alleles into supertypes would be an enormous feat.

While the current tools and data helped build up our current knowledge and give us a clear picture of how HLA molecules bind peptides, we are still far from being able to properly predict which peptides can successfully be presented by any given HLA. A step further will be to correlate peptide binding and immunogenicity, to not only enable our understanding of which peptide can be presented, but also which peptide bound HLAs will activate T cells.

This predictive ability will have enormous potential to help design better drugs to avoid drug hypersensitivity [62–66], as well as rapidly and accurately predict epitopes for newly emerging pathogens. A clear application that could help the current research of viral immunity, would be the prediction of SARS-CoV-2 peptides able to bind HLA molecules. A lot of the initial work on SARS-CoV-2 used peptide binding prediction, or prior knowledge of closely related viruses (i.e. SARS), to select relevant peptides to be studied. However, some early predictions failed to correctly identify HLA restriction for some peptides [68] or are only accurate at predicting a handful of the most common and well-studied HLA allotypes. Therefore, prediction for a larger number of HLA molecules would be highly informative and would help focus the study on selected peptides.

The holy grail of peptide prediction would be the ability to predict dominant immunogenic epitopes for a given HLA. Hopefully this can be achieved with widespread implementation and improvement of computational approaches using all data available (prediction, peptide affinity, mass spectrometry, T cell activation and pHLA structures). This information will be highly relevant for therapeutics such as peptide-based vaccine, and even developing personalized T cell-based therapy against pathogenic infections or cancer.

## Perspectives

- Understanding peptide binding specificity to HLA is key for developing T cell-based therapies such as vaccines.
- HLA grouping into supertypes is a quick and easy way to draw associations between HLA and peptide, but falls short to correctly help predicting peptide binding.
- Structural data integrated into the current predictive algorithm will improve peptide prediction and HLA association, with future algorithms able to predict immunogenicity as well.

## Open Access

Open access for this article was enabled by the participation of La Trobe University in an all-inclusive *Read & Publish* pilot with Portland Press and the Biochemical Society under a transformative agreement with CAUL.

## Abbreviations

HLA: Human leukocyte antigens
IEDB: Immune Epitope Database
MHC: major histocompatibility complex
PDB: Protein Data Bank
